# Analysis of Mortality and Morbidity in COVID-19 Patients with Obesity Using Clinical Epidemiological Data from the Korean Center for Disease Control & Prevention

**DOI:** 10.3390/ijerph17249336

**Published:** 2020-12-14

**Authors:** So Young Kim, Dae-Myoung Yoo, Chanyang Min, Jee Hye Wee, Joo-Hee Kim, Hyo Geun Choi

**Affiliations:** 1 Department of Otorhinolaryngology-Head & Neck Surgery, CHA University, Seongnam 13496, Korea; sossi81@hanmail.net; 2 Hallym Data Science Laboratory, Hallym University College of Medicine, Anyang 14068, Korea; ydm1285@naver.com (D.-M.Y.); joicemin@naver.com (C.M.); 3 Graduate School of Public Health, Seoul National University, Seoul 08826, Korea; 4 Department of Otorhinolaryngology-Head & Neck Surgery, Hallym University College of Medicine, Anyang 14068, Korea; weejh07@hanmail.net; 5 Allergy, and Critical Care medicine Department of Medicine, Hallym University Sacred Heart Hospital Hallym University College of Medicine, Anyang 14068, Korea; luxjhee@hallym.or.kr

**Keywords:** obesity, thinness, COVID-19, case-control studies, cohort studies, epidemiology

## Abstract

Previous studies have reported the association of obesity with increased morbidity or mortality due to the coronavirus disease 2019 (COVID-19). This study aims to investigate the relationship of obesity, as defined by the body mass index (BMI), with morbidity and mortality due to COVID-19. Data from 5628 confirmed COVID-19 patients were collected by the Centers for Disease Control and Prevention of Korea. The hazard ratios (HRs) for mortality in the BMI groups were analyzed using the Cox proportional hazard model adjusted for covariates. The odds ratios (ORs) of morbidity and diabetes in the BMI groups were analyzed using logistic regression adjusted for the same covariates. Both underweight and obesity were associated with a higher HR for mortality (adjusted HR = 2.28, 95% confidence intervals [95% CI] = 1.23–4.25, *p* = 0.009 for underweight and adjusted HR = 1.71, 95% CI = 1.10–2.66, *p* = 0.017 for obese). Obesity was related to higher odds of morbidity (adjusted OR = 1.71, 95% CI = 1.32–2.21, *p* < 0.001). Underweight and obesity were associated with high mortality and/or morbidity due to COVID-19 in Korea.

## 1. Introduction

Coronavirus disease 2019 (COVID-19) has been a global concern since December 2019, with >28.6 million affected patients and approximately 917,417 deaths worldwide as of 15 September 2020 [1]. The pathogen that causes COVID-19, severe acute respiratory syndrome coronavirus 2 (SARS-CoV-2), belongs to the Coronaviridae family, although it has distinct clinical features, and at least 41 RNA modification sites have been described [2,3]. Although the overall mortality rate is not especially high (3.2%), patients with severe COVID-19 experience a grave disease course that requires intensive care with mechanical ventilation and leads to chronic respiratory sequalae [4,5]. Because medical facilities and treatment resources are limited and the number of patients with COVID-19 is growing, the appropriate triage of patients with COVID-19 with the accurate prediction of disease severity and prognosis is necessary. Therefore, a large number of studies have investigated the factors associated with severe disease and a poor prognosis of COVID-19, including advanced age, high index for inflammation score, and comorbidities, such as cardiovascular disease, diabetes, respiratory disease and chronic kidney disease [6,7,8,9].

It has been suggested that obesity is a risk factor for severe COVID-19 [10,11,12]. Among < 50-year-old COVID-19 patients in the US, a body mass index (BMI) ≥ 40 kg/m^2^ was associated with a 5.1-fold higher mortality rate (95% CI = 2.3–11.1) [11]. Another retrospective study in the US demonstrated 3.78-fold higher in-hospital mortality rate among COVID-19 patients with BMI values ≥ 35 kg/m^2^ [12]. Reduced cardiorespiratory reserve and immune dysregulation were proposed as plausible mechanisms for the increased mortality due to COVID-19 in these severely obese patients [10]. Many studies have investigated severely obese patients [13]. However, the risk of mortality in patients with severe COVID-19 who are overweight or less severely obese is unclear [14,15]. Although obese patients have a higher rate of developing severe COVID-19 than nonobese patients, overweight patients did not have the same increased risk (odds ratio [OR] = 1.84, 95% CI = 0.99–3.43, *p* = 0.05) [14]. Another retrospective cohort study reported that the risk of severe COVID-19 was not significantly elevated in overweight and obese patients (BMI = 30–34.9), but was significantly elevated in severely obese patients (BMI ≥ 35) (OR = 5.39, 95% CI = 1.13–25.65, *p* = 0.034) [15]. In addition, the obesity paradox was proposed based on the decreased mortality due to acute respiratory distress syndrome (ARDS)/acute lung injury in patients with obesity [16]. It was suggested that preconditioning by obesity, which is a chronic inflammatory condition, might limit the adverse impacts of more serious inflammatory responses [17]. In summary, the impact of overweight and obesity on the severity of COVID-19 is unclear. Moreover, few studies have evaluated the association of underweight with the severity of COVID-19.

This study tested the hypothesis that an abnormal BMI, which includes underweight, overweight and obesity, might be associated with a poor prognosis of COVID-19. Mortality and morbidity related to COVID-19 were investigated in patients who were underweight, normal weight, overweight and obese.

## 2. Materials and Methods

### 2.1. Ethics

The ethics committee of Hallym University (2020–07-032) approved this study. The need to obtain written informed consent was waived by the Institutional Review Board. All analyses adhered to the guidelines and regulations of the ethics committee of Hallym University.

### 2.2. Study Population and Participant Selection

The clinical epidemiological data of all participants who were released from isolation by 30 April, 2020, after having recovered from confirmed cases of COVID-19, were analyzed. The data were collected by the Korea Centers for Disease Control and Prevention (KCDD). All included COVID-19 patients were confirmed by PCR for the SARS-CoV-2 positivity. Patients with confirmed cases of COVID-19 were released from isolation after achieving a complete recovery. Asymptomatic patients were determined to be completely recovered if the PCR results were negative two consecutive times with at least a 24-h interval between them at least seven days after a definitive diagnosis had been made. Symptomatic patients with confirmed cases were determined to be completely recovered if they had no fever without taking antipyretic drugs, the clinical manifestations were improved and the PCR results were negative two consecutive times with at least a 24-h interval between them at least seven days after a definitive diagnosis had been made.

**Figure 1:**
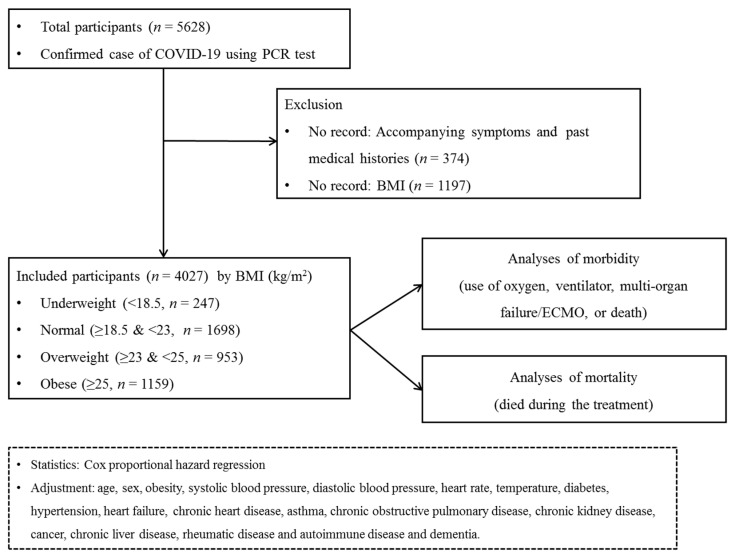
A schematic illustration of the participant selection process that was used in the present study. Of a total of 5628 patients with confirmed COVID-19, 247 underweight participants, 1698 normal participants, 953 overweight participants and 1159 obese participants were selected.

Participants who did not have records of symptoms or past medical histories were excluded (*n* = 374). Participants who did not have recorded BMI values were excluded (*n* = 1197). Finally, 247 underweight participants, 1698 normal weight participants, 953 overweight participants and 1159 obese participants were selected. (Figure 1).

### 2.3. Exposure (Obesity)

Obesity was measured using BMI (kg/m^2^). BMI was categorized as <18.5 (underweight), ≥18.5 to <23 (normal), ≥23 to <25 (overweight) and ≥25 (obese) based on the WHO cut-off values for the Asian population [18].

### 2.4. Outcome (Mortality)

During follow-up, mortality was recorded.

### 2.5. Outcome (Maximum Level of Morbidity)

The maximum level of morbidity during hospitalization was categorized as follows: no limitations on activity, limitations on activity but no supplemental oxygen needed, oxygen administered via a nasal cannula, oxygen administered via a facial mask, noninvasive ventilation, invasive ventilation, multiorgan failure/ECMO and death.

We divided the participants into groups based on their maximum level of morbidity during treatment: low morbidity (no limitations on activity, limitations on activity but no supplemental oxygen needed) and high morbidity (oxygen administered via a nasal cannula, oxygen administered via a facial mask, noninvasive ventilation, invasive ventilation, multiorgan failure/ECMO and death).

### 2.6. Covariates

Age groups were divided by 10-year intervals: 0–9, 10–19, 20–29… and 80+ years old (total of 9 age groups). Systolic blood pressure was divided into 5 groups: <120, 120–129, 130–139, 140–159 and ≥ 50 mmHg. Diastolic blood pressure was divided into 4 groups: <80, 80–89, 90–99 and ≥100 mmHg. Heart rate and temperature were measured. Missing systolic blood pressure and diastolic blood pressure [*n* = 33 (0.81%)] values were replaced by 120–129 and 80–89 mmHg, respectively, and missing values for temperature [*n* = 23 (0.56%)] and heart rate [*n* = 9 (0.22%)] were replaced by the mean value of each variable in the final selected participants.

The past medical histories of the following conditions were recorded: hypertension, heart failure, chronic heart disease, diabetes mellitus, asthma, chronic obstructive pulmonary disease, chronic kidney disease, cancer, chronic liver disease, rheumatic disease or autoimmune disease and dementia. The past medical histories were defined based on the international classification of diseases −10 codes.

### 2.7. Statistical Analyses

The general characteristics were compared among the underweight, normal weight, overweight and obese groups using the chi-square test for categorical variables and analysis of variance (ANOVA) for continuous variables.

To analyze the hazard ratios (HRs, i.e., the ratio of the rates of mortality due to COVID-19 compared to normal weight groups) with 95% confidence intervals (CIs) for mortality in the various BMI categories, a Cox proportional hazard regression model was used. In this analysis, crude and adjusted models (adjusted for age, sex, obesity, systolic blood pressure, diastolic blood pressure, heart rate, temperature, diabetes, hypertension, heart failure, chronic heart disease, asthma, chronic obstructive pulmonary disease, chronic kidney disease, cancer, chronic liver disease, rheumatic or autoimmune disease and dementia) were used.

To analyze the ORs (i.e., the ratio of the odds of morbidity/mortality of COVID-19 in the presence of each BMI category) of morbidity in the various BMI categories, a logistic regression model was used. In this analysis, crude and adjusted models (adjusted for age, sex, obesity, systolic blood pressure, diastolic blood pressure, heart rate, temperature, diabetes, hypertension, heart failure, chronic heart disease, asthma, chronic obstructive pulmonary disease, chronic kidney disease, cancer, chronic liver disease, rheumatic or autoimmune disease and dementia) were used. The reliability of these analyses were check by testing the joint significance of all predictors. (Tables S1 and S2)

For the subgroup analyses, we divided the participants to confirm the associations after stratification by age (< 50 years old and ≥ 50 years), sex and past medical histories. The division of the age groups was determined by the median value of all participants (Tables S3 and S4).

Two-tailed analyses were performed, and significance was defined as a *p*-value less than 0.05. SAS version 9.4 (SAS Institute Inc., Cary, NC, USA) was used for the statistical analyses.

## 3. Results

**Figure 2:**
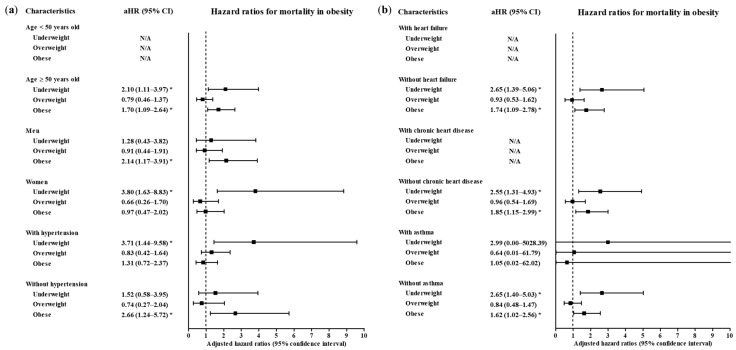
Subgroup analyses to obtain the adjusted hazard ratios (95% confidence interval) for mortality according to age, sex and hypertension (**a**) and heart failure, chronic heart failure and asthma (**b**). (* *p* < 0.05).

**Figure 3:**
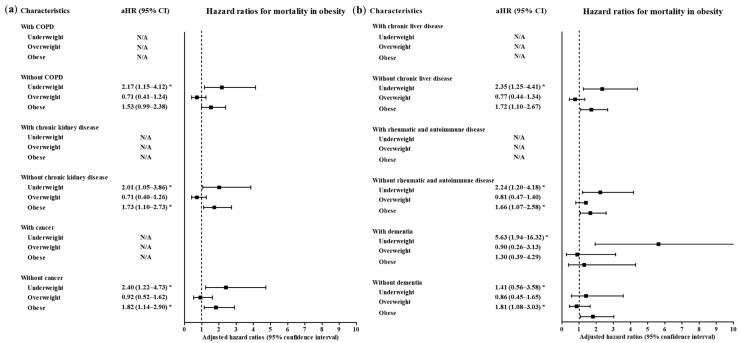
Subgroup analyses to obtain the adjusted hazard ratios (95% confidence interval) for mortality according to chronic obstructive pulmonary disease, chronic kidney disease and cancer (**a**) and chronic liver disease, rheumatic/autoimmune disease and dementia (**b**). (* *p* < 0.05).

A total of 4057 participants were classified as follows: 6.10% (247/4057) underweight, 41.85% (1698/4057) normal weight, 23.49% (953/4057) overweight and 28.57% (1159/4057) obese (Table 1). Age, sex, systolic blood pressure, diastolic blood pressure, heart rate and past medical histories of diabetes mellitus, hypertension, chronic obstructive pulmonary disease and dementia were different among the BMI groups (all *p* < 0.05). The mortality rates were 6.5% (213/4057), 2.7% (46/4057), 2.1% (20/4057) and 3.8% (44/4057) in the underweight, normal weight, overweight and obese groups, respectively (*p* = 0.002, Chi-square test). The rates of a high level of morbidity were 13.8% (34/4057), 11.3% (191/4057), 14.4% (137/4057) and 17.9% (207/4057) in the underweight, normal weight, overweight and obese groups, respectively (*p* < 0.001, Chi-square test). The associations of mortality due to COVID-19 with underweight and obesity were consistent in the subgroups of participants ≥ 50 years old, men, women, patients with and without hypertension, patients without heart failure, patients without chronic heart disease, patients with and without asthma, patients without chronic obstructive pulmonary disease (COPD), patients without chronic kidney disease, patients without cancer, patients without chronic liver disease, patients without rheumatic disease or autoimmune disease and patients with and without dementia (Figure 2 and Figure 3, and Table S1). Other subgroups could not be analyzed due to the small number of cases.

The underweight group had a 2.28-fold higher HR for mortality due to COVID-19 than the normal weight group (95% CI = 1.23–4.25, *p* = 0.009, Cox proportional hazard regression, adjusted model) (Table 2). Obese patients also had a higher HR for mortality due to COVID-19 than normal weight patients (adjusted HR = 1.71, 95% CI = 1.10–2.66, *p* = 0.017, Cox proportional hazard regression, adjusted model). Overweight patients did not differ in the risk of mortality due to COVID-19 when compared with normal weight patients (adjusted OR = 0.80, 95% CI = 0.46–1.39, *p* = 0.432, Cox proportional hazard regression, adjusted model).

**Figure 4:**
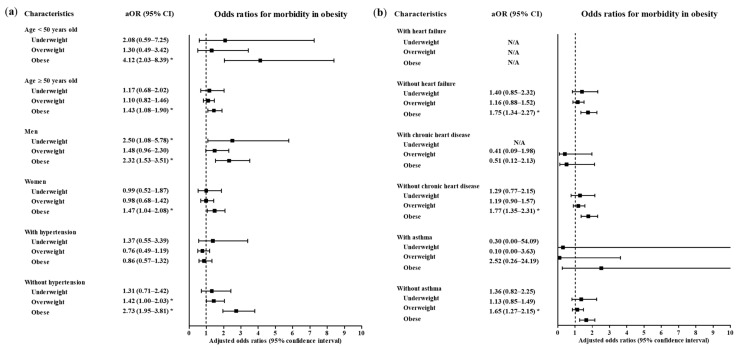
Subgroup analyses to obtain the adjusted odds ratios (95% confidence interval) for the maximum level of morbidity during hospitalization according to age, sex and hypertension (**a**) and heart failure, chronic heart disease and asthma (**b**). (* *p* < 0.05).

**Figure 5:**
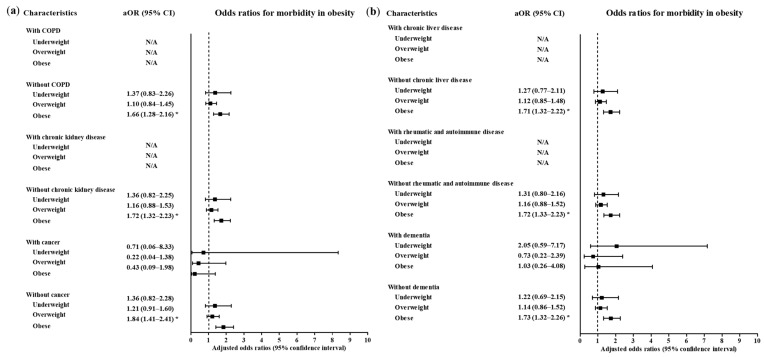
Subgroup analyses to obtain the adjusted odds ratios (95% confidence interval) for the maximum level of morbidity during hospitalization according to chronic obstructive pulmonary disease, chronic kidney disease and cancer (**a**) chronic liver disease, rheumatic and autoimmune disease and dementia (**b**). (* *p* < 0.05).

A high level of morbidity related to COVID-19 was also related to BMI (Table 3). Compared to the normal weight group, the obese group had higher odds of a high level of morbidity related to COVID-19 (adjusted OR = 1.71, 95% CI = 1.32–2.21, *p* < 0.001, Logistic regression model). The subgroups of patients who were <50 years old, ≥50 years old, males and females had consistent associations of obesity with a high morbidity level related to COVID-19 (Figure 4 and Figure 5, Table S2). In the subgroups without hypertension, without heart failure, without chronic heart disease, without asthma, without COPD, without chronic kidney disease, without cancer, without chronic liver disease, without rheumatic disease or autoimmune disease and without dementia, there were associations of obesity with a high level of morbidity due to COVID-19.

## 4. Discussion

Both underweight and obese individuals had increased risks of mortality due to COVID-19 in the Korean population. Obesity was also associated with the increased risk of morbidity of COVID-19. These associations of underweight and obesity with mortality and/or morbidity due to COVID-19 were consistent in all age and sex subgroups. Interestingly, these relations remained evident in the subgroups of patients without past medical histories. A number of recent studies have reported the potential risk of morbidity or mortality associated with COVID-19 in obese patients and patients with metabolic problems, such as diabetes, dyslipidemia and hypertension. This study adds to previous findings by demonstrating that underweight and obesity are associated with an increased risk of mortality and morbidity due to COVID-19. In addition, the present study demonstrated that the relationship of obesity with the risk of a poor prognosis of COVID-19 remained valid in an East Asian population, an ethnic group with a relatively lower proportion of morbidly obese individuals.

It has been suggested that obesity is related to high morbidity and/or mortality rates in COVID-19 patients in previous studies [9,13,19,20]. Obesity could be linked to the severity of COVID-19 via metabolic dysregulation, immune impairments and adiposopathy with altered adipokine levels, such as increased leptin and decreased adiponectin concentrations [13,19,21]. Obesity impairs the ability of the adaptive immune response to cope with viral infection [22]. AT synthesizes a number of pro-inflammatory adipokines and cytokines, including interleukin (IL)-6 and IL-8, which may weaken the antiviral immune response in obese patients [23]. Furthermore, the metabolic comorbidities of obese patients, such as diabetes, could contribute to the risk of developing severe COVID-19, although the present study adjusted for possible confounding metabolic diseases, including diabetes and hypertension. Prior studies reported higher COVID-19 morbidity or mortality rates in diabetic patients [9,24]. Diabetes was shown to be related to greater odds of poor early outcomes of COVID-19 (ICU admission, mechanical ventilation and mortality) at two weeks (adjusted OR = 2.02, 95% CI = 1.01–4.03 for mortality) in 450 patients with COVID-19 [9]. The hyperglycemic changes and metabolic disturbances in patients with diabetes might increase the severity of COVID-19 [25,26]. Hyperglycemia can both directly and indirectly hinder immune cell function by promoting the synthesis of oxidants and glycation products [26]. Moreover, the impaired immune response in diabetes patients has been shown to involve a decreased number of activated macrophages and decreased phagocytic activities compared to those in the healthy control group [25].

Underweight was related to mortality of COVID-19 in this study. To our knowledge, no previous study has assessed the association of underweight with COVID-19. However, the risk of viral infection in underweight patients has been described [27,28,29]. In an observational cohort study in Mexico, underweight adults had a 5.20-fold higher rate of influenza infection (95% CI = 1.67–16.01, *p* = 0.005) [27]. Impaired immunity due to protein malnutrition could increase the risk of viral infection in underweight patients [30]. An in vivo study demonstrated that mice given very-low-protein diets exhibited more severe influenza infections with a higher mortality rate and reduced antiviral antibody responses and levels of influenza nuclear protein-specific CD8+ T cells [30]. Thus, the impaired antiviral responses in underweight patients could be associated with the risk of severe COVID-19, as shown in the present study. In addition, the lack of inflammatory preconditioning, as seen in the obesity paradox in adults with ARDS, might partially explain the increased risk of severe COVID-19 in these patients [17]. In addition to ARDS, a prospective cohort study demonstrated that underweight patients had higher risk of respiratory disease-related mortality than normal weight patients (HR = 1.55, 95% CI = 1.32–1.83) [31]. To attenuate the possible confounding effects of underlying pulmonary diseases, asthma and COPD were adjusted for in this study.

This study revealed the relationship of an abnormal BMI with morbidity and mortality due to COVID-19 in a Korean national cohort. However, there are some limitations to consider when interpreting the results. Because of the retrospective study design, causality could not be determined in this study. The sample size was restricted, which could have resulted in insufficient statistical power. In the subgroup analyses, the morbidity and mortality in patients with past medical histories, such as heart failure, COPD, chronic kidney disease, cancer, chronic liver disease, rheumatic disease and autoimmune disease, could not be estimated. Although several potential confounders were adjusted for in this study, the possibility of some remaining confounders could not be excluded. For instance, histories of smoking and alcohol consumption were not available in this cohort data. For the diagnosis of diabetes, medication histories, types and severity of disease and blood glucose levels could not be assessed in this study. Because this study was based on a Korean population, ethnic differences could exist with regard to the association of BMI groups with the severity of and mortality due to COVID-19. In this study, the BMI groups were classified according to the WHO recommendations for the Asian population, which has lower reference values than those intended for use in Western countries [18]. Despite these limitations, the associations of obesity and underweight with severe COVID-19 could be clinically valuable information that could be used to stratify COVID-19 patients according to risk during the pandemic.

## 5. Conclusions

Both obesity and underweight were associated with high mortality and/or morbidity of COVID-19 in Korea. The patients without other comorbidities including hypertension, heart failure, chronic heart disease, asthma, COPD, chronic kidney disease, cancer, chronic liver disease, rheumatic and autoimmune disease and dementia demonstrated solid relations of obesity and/or underweight with mortality and/or morbidity of COVID-19.

## Figures and Tables

**Table 1:** General characteristics of the participants with COVID-19 according to obesity.

Characteristics	The Participants with COVID-19 ‡				
	Underweight	Normal	Overweight	Obese	*p*-Value
Total participants (*n*, %)	247 (100.0)	1668 (100.0)	953 (100.0)	1159 (100.0)	
Age (years old) (*n*, %)					<0.001 *
0–9	39 (15.8)	16 (0.9)	0 (0.0)	2 (0.2)	
10–19	27 (10.9)	86 (5.1)	25 (2.6)	37 (3.2)	
20–29	62 (25.1)	402 (23.7)	151 (15.8)	220 (19.0)	
30–39	22 (8.9)	170 (10.0)	96 (10.1)	148 (12.8)	
40–49	11 (4.5)	243 (14.3)	116 (12.2)	166 (14.3)	
50–59	20 (8.1)	313 (18.4)	220 (23.1)	248 (21.4)	
60–69	16 (6.5)	232 (13.7)	194 (20.4)	192 (16.6)	
70–79	28 (11.3)	136 (8.0)	109 (11.4)	101 (8.7)	
80+	22 (8.9)	100 (5.9)	42 (4.4)	45 (3.9)	
Sex (*n*, %)					<0.001 *
Male	84 (34.0)	571 (33.6)	455 (47.7)	613 (52.9)	
Female	163 (66.0)	1127 (66.4)	498 (52.3)	546 (47.1)	
Systolic blood pressure (*n*, %)					<0.001 *
<120 mmHg	104 (42.1)	541 (31.9)	180 (18.9)	171 (14.8)	
120–129 mmHg	59 (23.9)	384 (22.6)	213 (22.4)	244 (21.1)	
130–139 mmHg	35 (14.2)	322 (19.0)	197 (20.7)	257 (22.2)	
140–159 mmHg	32 (13.0)	340 (20.0)	264 (27.7)	363 (31.3)	
≥160 mmHg	17 (6.9)	111 (6.5)	99 (10.4)	124 (10.7)	
Diastolic blood pressure (*n*, %)					<0.001 *
<80 mmHg	128 (51.8)	770 (45.4)	316 (33.2)	318 (27.4)	
80–89 mmHg	71 (28.7)	576 (33.9)	336 (35.3)	423 (36.5)	
90–99 mmHg	32 (13.0)	248 (14.6)	203 (21.3)	276 (23.8)	
≥100 mmHg	16 (6.5)	104 (6.1)	98 (10.3)	142 (12.3)	
Heart rate (mean, SD)	89.28 (17.80)	84.11 (14.74)	85.30 (14.69)	86.41 (14.46)	<0.001 †
Temperature (mean, SD)	36.92 (0.54)	36.90 (0.54)	36.91 (0.53)	37.02 (0.58)	0.486
Past medical history					
Diabetes mellitus (*n*, %)	18 (7.3)	155 (9.1)	135 (14.2)	184 (15.9)	<0.001 *
Hypertension (*n*, %)	28 (11.3)	228 (13.4)	237 (24.9)	336 (29.0)	<0.001 *
Heart failure (*n*, %)	6 (2.4)	12 (0.7)	8 (0.8)	14 (1.2)	0.060
Chronic heart disease (*n*, %)	7 (2.8)	41 (2.4)	40 (4.2)	44 (3.8)	0.052
Asthma (*n*.%)	5 (2.0)	30 (1.8)	31 (3.3)	30 (2.6)	0.100
COPD (*n*, %)	6 (2.4)	14 (0.8)	5 (0.5)	5 (0.4)	0.008*
Chronic kidney disease (*n*, %)	3 (1.2)	17 (1.0)	8 (0.8)	15 (1.3)	0.763
Cancer (*n*, %)	7 (2.8)	48 (2.8)	26 (2.7)	26 (2.2)	0.800
Chronic liver disease (*n*, %)	3 (1.2)	19 (1.1)	15 (1.6)	21 (1.8)	0.460
Rheumatic or autoimmune disease (*n*, %)	1 (0.4)	18 (1.1)	8 (0.8)	4 (0.4)	0.162
Dementia (*n*, %)	20 (8.1)	64 (3.7)	20 (2.1)	16 (1.4)	<0.001 *
Death (*n*, %)	16 (6.5)	46 (2.7)	20 (2.1)	44 (3.8)	0.002 *
Maximum morbidity					<0.001 *
Low morbidity	213 (86.2)	1507 (88.8)	816 (85.6)	952 (82.1)	
High morbidity	34 (13.8)	191 (11.3)	137 (14.4)	207 (17.9)	

Abbreviations: COPD, chronic obstructive pulmonary disease. * Chi-square test. Significance at *p* < 0.05. † Analysis of variance (ANOVA). Significance at *p* < 0.05. ‡ Obesity (BMI, body mass index, kg/m2) was categorized as <18.5 (underweight), ≥18.5 to <23 (normal), ≥23 to <25 (overweight), ≥25 to <30 (obese I) and ≥30 (obese II).

**Table 2:** Crude and adjusted hazard ratios (95% confidence interval) of obesity for mortality.

Obesity (BMI, kg/m^2^)	HRs for Death			
	Crude	*p*-Value	Adjusted †	*p*-Value
Underweight (<18.5)	2.56 (1.45–4.52)	0.001 *	2.28 (1.23–4.25)	0.009 *
Normal (≥18.5 to <23)	1		1	
Overweight (≥23 to <25)	0.79 (0.47–1.34)	0.387	0.80 (0.46–1.39)	0.432
Obese (≥25)	1.36 (0.90–2.05)	0.149	1.71 (1.10–2.66)	0.017 *

* Cox proportional hazard regression model, Significance at *p* < 0.05. † The model was adjusted for age, sex, obesity, systolic blood pressure, diastolic blood pressure, heart rate, temperature, diabetes, hypertension, heart failure, chronic heart disease, asthma, chronic obstructive pulmonary disease, chronic kidney disease, cancer, chronic liver disease, rheumatic or autoimmune disease and dementia.

**Table 3:** Crude and adjusted odd ratios (95% confidence interval) of obesity for maximum morbidity score during hospitalization.

Obesity (BMI, kg/m^2^)	ORs for High Morbidity			
	Crude	*p*-Value	Adjusted †	*p*-Value
Underweight (<18.5)	1.26 (0.85–1.86)	0.249	1.29 (0.79–2.12)	0.312
Normal (≥18.5 to <23)	1		1	
Overweight (≥23 to <25)	1.33 (1.05–1.68)	0.019 *	1.13 (0.86–1.49)	0.369
Obese (≥25)	1.72 (1.39–2.12)	<0.001 *	1.71 (1.32–2.21)	<0.001 *

* Logistic regression model, Significance at *p* < 0.05. † The model was adjusted for age, sex, obesity, systolic blood pressure, diastolic blood pressure, heart rate, temperature, diabetes, hypertension, heart failure, chronic heart disease, asthma, chronic obstructive pulmonary disease, chronic kidney disease, cancer, chronic liver disease, rheumatic or autoimmune disease and dementia.

## Supplementary Materials

The following are available online at https://www.mdpi.com/1660-4601/17/24/9336/s1, Table S1 Subgroup analyses of crude and adjusted hazard ratios (95% confidence interval) for death in obesity and diabetes, Table S2 Subgroup analyses of crude and adjusted odds ratios (95% confidence interval) for treatment in obesity and diabetes. Table S3 Subgroup analyses of crude and adjusted hazard ratios (95% confidence interval) for mortality in obesity. Table S4 Subgroup analyses of crude and adjusted odds ratios (95% confidence interval) for maximum morbidity score during hospitalization.

## Abbreviations

COVID-19: coronavirus disease 2019; BMI, body mass index; HRs, hazard ratios; 95% CI, 95% confidence intervals; SARS-CoV-2, severe acute respiratory syndrome coronavirus 2; OR, odds ratio; ARDS, acute respiratory distress syndrome; ANOVA, analysis of variance; HRs, hazard ratios; COPD, chronic obstructive pulmonary disease; SD, standard deviation.
