# Supplementary material for: Analysis of Mortality and Morbidity in COVID-19 Patients with Obesity Using Clinical Epidemiological Data from the Korean Center for Disease Control & Prevention
Source: Int J Environ Res Public Health. 2020 Dec 14;17(24):9336. doi: 10.3390/ijerph17249336 (PMC7764883; doi:10.3390/ijerph17249336)
Supplement: Supplementary file 1 [file ijerph-17-09336-s001.pdf]

Table S1Testing the joint significance of all predictors for mortality by obesity in Cox regression.

Test	Chi-Square	DF	P-value
Likelihood Ratio	436.78	25	<0.001
Score	589.99	25	<0.001
Wald	276.21	25	<0.001

Table S2 Testing the joint significance of all predictors for morbidity by obesity in Logistic regression.

Test	Chi-Square	DF	P-value
Likelihood Ratio	885.43	25	<0.001
Score	884.37	25	<0.001
Wald	572.73	25	<0.001

Table S3 Subgroup analyses of crude and adjusted hazard ratios (95% confidence interval) for mortality in obesity.

**Characteristics**	**Hazard ratios for death**			
	**Crude**	**P-value**	Adjusted†	P-value
**Age < 50 years old (n = 2,039)**				
Underweight (< 18.5)	1		1	
Normal (≥ 18.5 to < 23)	N/A		N/A	
Overweight (≥ 23 to < 25)	N/A		N/A	
Obese (≥ 25)	N/A		N/A	
**Age ≥ 50 years old (n = 2,018)**				
Underweight (< 18.5)	3.07 (1.72-5.51)	<0.001*	2.10 (1.11-3.97)	0.022*
Normal (≥ 18.5 to < 23)	1		1	
Overweight (≥ 23 to < 25)	0.62 (0.36-1.04)	0.070	0.79 (0.46-1.37)	0.400
Obese (≥ 25)	1.26 (0.84-1.91)	0.268	1.70 (1.09-2.64)	0.018*
**Men (n =1,723)**				
Underweight (< 18.5)	1.68 (0.64-4.43)	0.294	1.28 (0.43-3.82)	0.657
Normal (≥ 18.5 to < 23)	1		1	
Overweight (≥ 23 to < 25)	0.83 (0.43-1.61)	0.576	0.91 (0.44-1.91)	0.810
Obese (≥ 25)	1.27 (0.74-2.18)	0.395	2.14 (1.17-3.91)	0.013*
**Women (n = 2,334)**				
Underweight (< 18.5)	3.42 (1.67-7.02)	<0.001*	3.80 (1.63-8.83)	0.002*
Normal (≥ 18.5 to < 23)	1		1	
Overweight (≥ 23 to < 25)	0.58 (0.24-1.43)	0.239	0.66 (0.26-1.70)	0.393
Obese (≥ 25)	1.14 (0.59-2.23)	0.694	0.97 (0.47-2.02)	0.941
**With hypertension (n = 829)**				
Underweight (< 18.5)	2.59 (1.22-5.49)	0.013*	3.71 (1.44-9.58)	0.007*
Normal (≥ 18.5 to < 23)	1		1	
Overweight (≥ 23 to < 25)	0.49 (0.26-0.92)	0.028*	0.83 (0.42-1.64)	0.596
Obese (≥ 25)	0.62 (0.37-1.07)	0.084	1.31 (0.72-2.37)	0.375
**Without hypertension (n = 3,228)**				
Underweight (< 18.5)	3.10 (1.28-7.49)	0.012*	1.52 (0.58-3.95)	0.393
Normal (≥ 18.5 to < 23)	1		1	
Overweight (≥ 23 to < 25)	0.76 (0.30-1.92)	0.558	0.74 (0.27-2.04)	0.560
Obese (≥ 25)	1.92 (1.00-3.69)	0.052	2.66 (1.24-5.72)	0.012*
**With heart failure (n = 40)**				
Underweight (< 18.5)	1.63 (0.27-9.79)	0.594	N/A	
Normal (≥ 18.5 to < 23)	1		1	
Overweight (≥ 23 to < 25)	N/A		N/A	
Obese (≥ 25)	1.67 (0.40-7.03)	0.482	N/A	
**Without heart failure (n = 4017)**				
Underweight (< 18.5)	2.45 (1.34-4.48)	0.004*	2.65 (1.39-5.06)	0.003*
Normal (≥ 18.5 to < 23)	1		1	
Overweight (≥ 23 to < 25)	0.85 (0.50-1.44)	0.544	0.93 (0.53-1.62)	0.795

Obese (≥ 25)	1.29 (0.83-1.98)	0.258	1.74 (1.09-2.78)	0.021*
**With chronic heart disease (n = 132)**				
Underweight (< 18.5)	1.83 (0.38-8.85)	0.453	N/A	
Normal (≥ 18.5 to < 23)	1		1	
Overweight (≥ 23 to < 25)	0.15 (0.02-1.25)	0.080	N/A	
Obese (≥ 25)	0.50 (0.15-1.67)	0.259	N/A	
**Without chronic heart disease (n = 3,925)**				
Underweight (< 18.5)	2.71 (1.47-5.00)	0.001*	2.55 (1.31-4.93)	0.006*
Normal (≥ 18.5 to < 23)	1		1	
Overweight (≥ 23 to < 25)	0.92 (0.53-1.60)	0.779	0.96 (0.54-1.69)	0.876
Obese (≥ 25)	1.50 (0.96-2.35)	0.072	1.85 (1.15-2.99)	0.012*
**With asthma (n = 96)**				
Underweight (< 18.5)	3.58 (0.32-39.84)	0.300	2.99 (0.00-5028.39)	0.773
Normal (≥ 18.5 to < 23)	1		1	
Overweight (≥ 23 to < 25)	N/A		0.64 (0.01-61.79)	0.849
Obese (≥ 25)	1.43 (0.24-8.60)	0.695	1.05 (0.02-62.02)	0.981
**Without asthma (n = 3,961)**				
Underweight (< 18.5)	2.52 (1.40-4.53)	0.002*	2.65 (1.40-5.03)	0.003*
Normal (≥ 18.5 to < 23)	1		1	
Overweight (≥ 23 to < 25)	0.84 (0.50-1.43)	0.530	0.84 (0.48-1.47)	0.550
Obese (≥ 25)	1.29 (0.84-1.98)	0.254	1.62 (1.02-2.56)	0.040*
**With chronic obstructive pulmonary disease (n = 30)**				
Underweight (< 18.5)	N/A		N/A	
Normal (≥ 18.5 to < 23)	1		1	
Overweight (≥ 23 to < 25)	N/A		N/A	
Obese (≥ 25)	N/A		N/A	
**Without chronic obstructive pulmonary disease (n = 4,027)**				
Underweight (< 18.5)	2.44 (1.36-4.37)	0.003*	2.17 (1.15-4.12)	0.017*
Normal (≥ 18.5 to < 23)	1		1	
Overweight (≥ 23 to < 25)	0.75 (0.44-1.28)	0.287	0.71 (0.41-1.24)	0.226
Obese (≥ 25)	1.28 (0.84-1.95)	0.244	1.53 (0.99-2.38)	0.058
**With chronic kidney disease (n = 43)**				
Underweight (< 18.5)	2.32 (0.25-22.01)	0.462	N/A	
Normal (≥ 18.5 to < 23)	1		1	
Overweight (≥ 23 to < 25)	1.77 (0.30-10.38)	0.527	N/A	
Obese (≥ 25)	1.05 (0.23-4.71)	0.954	N/A	
**Without chronic kidney disease (n = 4,014)**				
Underweight (< 18.5)	2.63 (1.46-4.74)	0.001*	2.01 (1.05-3.86)	0.035*
Normal (≥ 18.5 to < 23)	1		1	
Overweight (≥ 23 to < 25)	0.78 (0.45-1.35)	0.372	0.71 (0.40-1.26)	0.239
Obese (≥ 25)	1.39 (0.90-2.13)	0.137	1.73 (1.10-2.73)	0.019*
**With cancer (n = 107)**				
Underweight (< 18.5)	1.83 (0.38-8.83)	0.451	N/A	
Normal (≥ 18.5 to < 23)	1		1	
Overweight (≥ 23 to < 25)	N/A		N/A	
Obese (≥ 25)	0.91 (0.23-3.53)	0.891	N/A	
**Without cancer (n = 3,950)**				
Underweight (< 18.5)	2.72 (1.47-5.02)	0.001*	2.40 (1.22-4.73)	0.011*
Normal (≥ 18.5 to < 23)	1		1	
Overweight (≥ 23 to < 25)	0.96 (0.56-1.65)	0.885	0.92 (0.52-1.62)	0.774
Obese (≥ 25)	1.52 (0.98-2.36)	0.063	1.82 (1.14-2.90)	0.013*
**With chronic liver disease (n = 58)**				
Underweight (< 18.5)	N/A	0.000	N/A	
Normal (≥ 18.5 to < 23)	1		1	
Overweight (≥ 23 to < 25)	N/A	0.000	N/A	
Obese (≥ 25)	N/A	0.000	N/A	
**Without chronic liver disease (n = 3,999)**				
Underweight (< 18.5)	2.47 (1.38-4.42)	0.002*	2.35 (1.25-4.41)	0.008*
Normal (≥ 18.5 to < 23)	1		1	
Overweight (≥ 23 to < 25)	0.76 (0.44-1.29)	0.308	0.77 (0.44-1.34)	0.349
Obese (≥ 25)	1.33 (0.88-2.01)	0.181	1.72 (1.10-2.67)	0.0174*
**With rheumatic disease and autoimmune disease (n = 31)**				

Underweight (< 18.5)	N/A		N/A	
Normal (≥ 18.5 to < 23)	1		1	
Overweight (≥ 23 to < 25)	N/A		N/A	
Obese (≥ 25)	N/A		N/A	
**Without rheumatic disease and autoimmune disease (n = 4,026)**				
Underweight (< 18.5)	2.54 (1.44-4.49)	0.001*	2.24 (1.20-4.18)	0.011*
Normal (≥ 18.5 to < 23)	1		1	
Overweight (≥ 23 to < 25)	0.79 (0.47-1.34)	0.390	0.81 (0.47-1.40)	0.449
Obese (≥ 25)	1.32 (0.87-2.00)	0.196	1.66 (1.07-2.58)	0.025*
**With dementia (n = 120)**				
Underweight (< 18.5)	3.01 (1.28-7.09)	0.012*	5.63 (1.94-16.32)	0.002*
Normal (≥ 18.5 to < 23)	1		1	
Overweight (≥ 23 to < 25)	1.24 (0.45-3.43)	0.685	0.90 (0.26-3.13)	0.869
Obese (≥ 25)	2.23 (0.85-5.86)	0.104	1.30 (0.39-4.29)	0.668
**Without dementia (n = 3,937)**				
Underweight (< 18.5)	1.69 (0.74-3.84)	0.210	1.41 (0.56-3.58)	0.470
Normal (≥ 18.5 to < 23)	1		1	
Overweight (≥ 23 to < 25)	0.86 (0.46-1.59)	0.628	0.86 (0.45-1.65)	0.655
Obese (≥ 25)	1.66 (1.04-2.68)	0.036*	1.81 (1.08-3.03)	0.024*

Abbreviation: N/A, Not applicable.

* Cox proportional hazard regression model, Significance at P < 0.05.

† The model was adjusted for age, sex, obesity, systolic blood pressure, diastolic blood pressure, heart rate, temperature, diabetes, hypertension, heart failure, chronic heart disease, asthma, chronic obstructive pulmonary disease, chronic kidney disease, cancer, chronic liver disease, rheumatic or autoimmune disease, and dementia.

Table S4 Subgroup analyses of crude and adjusted odds ratios (95% confidence interval) for maximum morbidity score during hospitalization.

**Obesity (BMI, kg/m^2)**	**ORs for high morbidity**			
	**Crude**	**P-value**	Adjusted†	P-value
**Age < 50 years old (n = 2,039)**				
Underweight (< 18.5)	1.64 (0.53-5.06)	0.386	2.08 (0.59-7.25)	0.253
Normal (≥ 18.5 to < 23)	1		1	
Overweight (≥ 23 to < 25)	1.19 (0.48-2.96)	0.716	1.30 (0.49-3.42)	0.597
Obese (≥ 25)	4.58 (2.46-8.53)	<0.001*	4.12 (2.03-8.39)	<0.001*
**Age ≥ 50 years old (n = 2,018)**				
Underweight (< 18.5)	1.83 (1.14-2.94)	0.013*	1.17 (0.68-2.02)	0.574
Normal (≥ 18.5 to < 23)	1		1	
Overweight (≥ 23 to < 25)	1.02 (0.79-1.32)	0.882	1.10 (0.82-1.46)	0.539
Obese (≥ 25)	1.38 (1.08-1.77)	0.010*	1.43 (1.08-1.90)	0.013*
**Men (n =1,723)**				
Underweight (< 18.5)	1.68 (0.93-3.07)	0.088	2.50 (1.08-5.78)	0.032*
Normal (≥ 18.5 to < 23)	1		1	
Overweight (≥ 23 to < 25)	1.39 (0.98-1.98)	0.067	1.48 (0.96-2.30)	0.078
Obese (≥ 25)	1.69 (1.23-2.33)	0.001*	2.32 (1.53-3.51)	<0.001*
**Women (n = 2,334)**				
Underweight (< 18.5)	1.03 (0.61-1.74)	0.906	0.99 (0.52-1.87)	0.962
Normal (≥ 18.5 to < 23)	1		1	
Overweight (≥ 23 to < 25)	1.20 (0.87-1.67)	0.262	0.98 (0.68-1.42)	0.929
Obese (≥ 25)	1.64 (1.22-2.20)	0.001*	1.47 (1.04-2.08)	0.031*
**With hypertension (n = 829)**				
Underweight (< 18.5)	1.43 (0.65-3.15)	0.374	1.37 (0.55-3.39)	0.502
Normal (≥ 18.5 to < 23)	1		1	
Overweight (≥ 23 to < 25)	0.61 (0.41-0.90)	0.014*	0.76 (0.49-1.19)	0.235
Obese (≥ 25)	0.62 (0.44-0.89)	0.010*	0.86 (0.57-1.32)	0.498
**Without hypertension (n = 3,228)**				
Underweight (< 18.5)	1.38 (0.84-2.25)	0.200	1.31 (0.71-2.42)	0.394
Normal (≥ 18.5 to < 23)	1		1	

Overweight (≥ 23 to < 25)	1.48 (1.08-2.02)	0.015*	1.42 (1.00-2.03)	0.053
Obese (≥ 25)	2.11 (1.60-2.79)	<0.001*	2.73 (1.95-3.81)	<0.001*
**With heart failure (n = 40)**				
Underweight (< 18.5)	0.71 (0.10-5.12)	0.738	N/A	
Normal (≥ 18.5 to < 23)	1		1	
Overweight (≥ 23 to < 25)	1.19 (0.19-7.46)	0.852	N/A	
Obese (≥ 25)	0.95 (0.20-4.54)	0.951	N/A	
**Without heart failure (n = 4017)**				
Underweight (< 18.5)	1.21 (0.80-1.81)	0.369	1.40 (0.85-2.32)	0.187
Normal (≥ 18.5 to < 23)	1		1	
Overweight (≥ 23 to < 25)	1.33 (1.04-1.68)	0.021*	1.16 (0.88-1.52)	0.309
Obese (≥ 25)	1.72 (1.38-2.13)	<0.001*	1.75 (1.34-2.27)	<0.001*
**With chronic heart disease (n = 132)**				
Underweight (< 18.5)	1.06 (0.21-5.35)	0.945	N/A	
Normal (≥ 18.5 to < 23)	1		1	
Overweight (≥ 23 to < 25)	0.54 (0.21-1.36)	0.189	0.41 (0.09-1.98)	0.269
Obese (≥ 25)	0.66 (0.27-1.60)	0.357	0.51 (0.12-2.13)	0.357
**Without chronic heart disease (n = 3,925)**				
Underweight (< 18.5)	1.26 (0.84-1.90)	0.261	1.29 (0.77-2.15)	0.339
Normal (≥ 18.5 to < 23)	1		1	
Overweight (≥ 23 to < 25)	1.37 (1.07-1.74)	0.013*	1.19 (0.90-1.57)	0.236
Obese (≥ 25)	1.78 (1.43-2.23)	<0.001*	1.77 (1.35-2.31)	<0.001*
**With asthma (n = 96)**				
Underweight (< 18.5)	1.25 (0.11-13.68)	0.855	0.30 (0.00-54.09)	0.649
Normal (≥ 18.5 to < 23)	1		1	
Overweight (≥ 23 to < 25)	0.96 (0.25-3.73)	0.955	0.10 (0.00-3.63)	0.211
Obese (≥ 25)	3.33 (1.00-11.14)	0.051	2.52 (0.26-24.19)	0.423
**Without asthma (n = 3,961)**				
Underweight (< 18.5)	1.26 (0.85-1.87)	0.258	1.36 (0.82-2.25)	0.229
Normal (≥ 18.5 to < 23)	1		1	
Overweight (≥ 23 to < 25)	1.33 (1.05-1.69)	0.019*	1.13 (0.85-1.49)	0.400
Obese (≥ 25)	1.66 (1.34-2.07)	<0.001*	1.65 (1.27-2.15)	<0.001*
**With chronic obstructive pulmonary disease (n = 30)**				
Underweight (< 18.5)	1.80 (0.26-12.50)	0.552	N/A	
Normal (≥ 18.5 to < 23)	1		1	
Overweight (≥ 23 to < 25)	7.20 (0.62-83.34)	0.114	N/A	
Obese (≥ 25)	7.20 (0.62-83.34)	0.114	N/A	
**Without chronic obstructive pulmonary disease (n = 4,027)**				
Underweight (< 18.5)	1.19 (0.79-1.79)	0.404	1.37 (0.83-2.26)	0.224
Normal (≥ 18.5 to < 23)	1		1	
Overweight (≥ 23 to < 25)	1.31 (1.04-1.67)	0.025*	1.10 (0.84-1.45)	0.497
Obese (≥ 25)	1.72 (1.39-2.13)	<0.001*	1.66 (1.28-2.16)	<0.001*
**With chronic kidney disease (n = 43)**				
Underweight (< 18.5)	0.44 (0.03-5.88)	0.538	N/A	
Normal (≥ 18.5 to < 23)	1		1	
Overweight (≥ 23 to < 25)	0.53 (0.10-2.98)	0.474	N/A	
Obese (≥ 25)	1.78 (0.42-7.47)	0.432	N/A	
**Without chronic kidney disease (n = 4,014)**				
Underweight (< 18.5)	1.29 (0.87-1.92)	0.212	1.36 (0.82-2.25)	0.232
Normal (≥ 18.5 to < 23)	1		1	
Overweight (≥ 23 to < 25)	1.36 (1.07-1.73)	0.012*	1.16 (0.88-1.53)	0.297
Obese (≥ 25)	1.71 (1.38-2.13)	<0.001*	1.72 (1.32-2.23)	<0.001*
**With cancer (n = 107)**				
Underweight (< 18.5)	0.73 (0.13-4.17)	0.723	0.71 (0.06-8.33)	0.788
Normal (≥ 18.5 to < 23)	1		1	
Overweight (≥ 23 to < 25)	0.33 (0.10-1.12)	0.076	0.22 (0.04-1.38)	0.106
Obese (≥ 25)	0.67 (0.24-1.92)	0.458	0.43 (0.09-1.98)	0.278
**Without cancer (n = 3,950)**				
Underweight (< 18.5)	1.31 (0.87-1.96)	0.197	1.36 (0.82-2.28)	0.239
Normal (≥ 18.5 to < 23)	1		1	
Overweight (≥ 23 to < 25)	1.42 (1.12-1.81)	0.004*	1.21 (0.91-1.60)	0.186
Obese (≥ 25)	1.82 (1.46-2.26)	<0.001*	1.84 (1.41-2.41)	<0.001*

**With chronic liver disease (n = 58)**				
Underweight (< 18.5)	7.50 (0.53-105.27)	0.135	N/A	
Normal (≥ 18.5 to < 23)	1		1	
Overweight (≥ 23 to < 25)	0.94 (0.18-5.02)	0.940	N/A	
Obese (≥ 25)	1.88 (0.45-7.82)	0.388	N/A	
**Without chronic liver disease (n = 3,999)**				
Underweight (< 18.5)	1.20 (0.81-1.80)	0.364	1.27 (0.77-2.11)	0.349
Normal (≥ 18.5 to < 23)	1		1	
Overweight (≥ 23 to < 25)	1.33 (1.05-1.69)	0.019*	1.12 (0.85-1.48)	0.406
Obese (≥ 25)	1.70 (1.37-2.11)	<0.001*	1.71 (1.32-2.22)	<0.001*
**With rheumatic disease and autoimmune disease (n = 31)**				
Underweight (< 18.5)	N/A		N/A	
Normal (≥ 18.5 to < 23)	1		1	
Overweight (≥ 23 to < 25)	0.37 (0.04-3.84)	0.406	N/A	
Obese (≥ 25)	0.87 (0.07-10.42)	0.910	N/A	
**Without rheumatic disease and autoimmune disease (n = 4,026)**				
Underweight (< 18.5)	1.29 (0.87-1.91)	0.206	1.31 (0.80-2.16)	0.285
Normal (≥ 18.5 to < 23)	1		1	
Overweight (≥ 23 to < 25)	1.35 (1.07-1.71)	0.013*	1.16 (0.88-1.52)	0.306
Obese (≥ 25)	1.74 (1.41-2.16)	<0.001*	1.72 (1.33-2.23)	<0.001*
**With dementia (n = 120)**				
Underweight (< 18.5)	1.39 (0.51-3.80)	0.527	2.05 (0.59-7.17)	0.260
Normal (≥ 18.5 to < 23)	1		1	
Overweight (≥ 23 to < 25)	1.13 (0.42-3.10)	0.807	0.73 (0.22-2.39)	0.604
Obese (≥ 25)	1.46 (0.48-4.39)	0.504	1.03 (0.26-4.08)	0.968
**Without dementia (n = 3,937)**				
Underweight (< 18.5)	1.03 (0.65-1.64)	0.895	1.22 (0.69-2.15)	0.499
Normal (≥ 18.5 to < 23)	1		1	
Overweight (≥ 23 to < 25)	1.44 (1.13-1.85)	0.004*	1.14 (0.86-1.52)	0.365
Obese (≥ 25)	1.92 (1.53-2.40)	<0.001*	1.73 (1.32-2.26)	<0.001*

Abbreviation: N/A, Not applicable.

* Logistic regression model, Significance at P < 0.05.

† The model was adjusted for age, sex, obesity, systolic blood pressure, diastolic blood pressure, heart rate, temperature, diabetes, hypertension, heart failure, chronic heart disease, asthma, chronic obstructive pulmonary disease, chronic kidney disease, cancer, chronic liver disease, rheumatic or autoimmune disease, and dementia.
